# Case Report of a Neonate with Complex Gastroschisis: A Multidisciplinary Approach

**DOI:** 10.3390/pediatric16030065

**Published:** 2024-09-09

**Authors:** Palanikumar Balasundaram, Timothy B. Lautz, Rhonda Gale, Kimberly G. Remedios-Smith

**Affiliations:** 1 Division of Neonatology, Department of Pediatrics, Mercy Health—Javon Bea Hospital, Rockford, IL 61114, USA; rgale@mhemail.org (R.G.); kremediossmith@mhemail.org (K.G.R.-S.); 2 Division of Pediatric Surgery, Department of Surgery, Ann & Robert H. Lurie Children’s Hospital of Chicago, Chicago, IL 60611, USA; tlautz@luriechildrens.org

**Keywords:** gastroschisis, vanishing gastroschisis, complex gastroschisis, neonate

## Abstract

Gastroschisis is a congenital anomaly characterized by herniation of abdominal contents via a defect in the anterior abdominal wall. Gastroschisis can manifest as simple or complex, with additional complications such as atresia, perforation, ischemia, necrosis, or volvulus. While prenatal screening and advancements in surgical techniques have improved outcomes, infants with complex gastroschisis cases pose significant challenges in neonatal care. Vanishing gastroschisis, a rare but dreaded complication with a mortality rate ranging from 10 to 70%, occurs when the abdominal wall closes around the herniated bowel, leading to strangulation. We present a case report focusing on the management of neonatal gastroschisis in a 36-week-old female infant with vanishing gastroschisis. The infant’s clinical course, including surgical interventions, complications, and multidisciplinary management, is discussed in detail. This case underscores the importance of a multidisciplinary approach in optimizing outcomes for infants with complex gastroschisis. Via this case report, we aim to provide insights into the complexities of neonatal gastroschisis management and advocate for a collaborative approach involving neonatology, pediatric surgery, infectious disease, and palliative care to improve outcomes and quality of life for affected infants.

## 1. Introduction

Gastroschisis, a term coined by Tarufi in 1894, originates from the Greek words “gastro”, meaning stomach, and “schisis”, indicating a split [1]. However, it is a misnomer as the anterior abdominal wall, not the stomach, is the site of the split. Various embryologic hypotheses elucidate the etiology of gastroschisis, including anomalous involution of the right umbilical vein, failure of embryonic mesenchyme differentiation induced by teratogenic exposure, amniotic membrane rupture at the umbilical cord base, interruption of the omphalomesenteric artery leading to localized necrosis, or abnormalities in the rudimentary umbilical ring [2]. Gastroschisis can manifest as simple or complex, with additional complications such as atresia, perforation, ischemia, necrosis, or volvulus [3]. We present a case report focusing on the management of neonatal gastroschisis in a 36-week-old gestational preterm female infant with vanishing gastroschisis. Via this case study, we aim to provide insights into the complexities of neonatal gastroschisis management and advocate for a collaborative approach involving neonatology, pediatric surgery, infectious disease, and palliative care to improve outcomes and quality of life for affected infants.

## 2. Case Presentation

**Figure 1:**
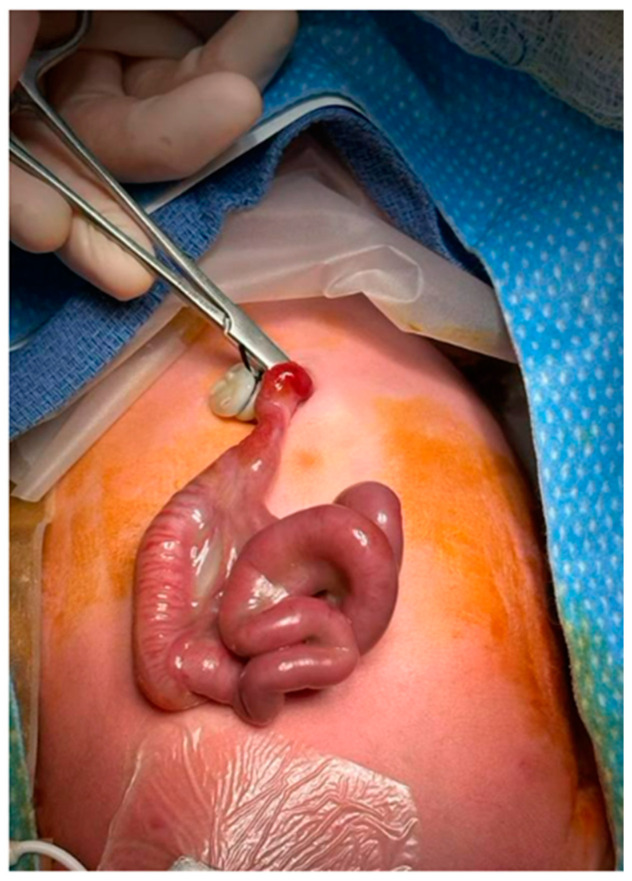
Admission examination showing vanishing gastroschisis.

A 36-week female infant was born to a 23-year-old mother via vaginal delivery after induction of labor due to intrauterine growth restriction (11th percentile) and elevated Dopplers. Prenatal care was adequate, with antenatal ultrasounds revealing fetal gastroschisis. The infant’s APGAR scores at 1 and 5 min were 8 and 9, respectively, with routine care initiated upon birth. On examination, the infant had a very narrow gastroschisis defect with an extrusion of a short loop of intestine (Figure 1).

**Figure 2:**
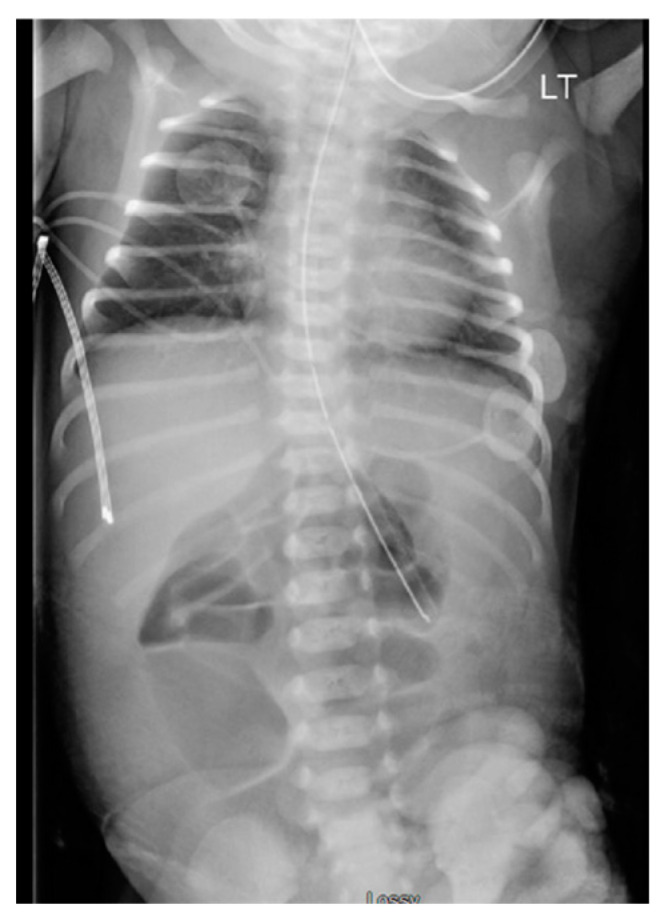
Day 1 abdominal X-ray revealing dilated intestines.

Initial management involved placing sterile saline-soaked gauze on the exposed bowel and wrapping it with a sterile bag. The infant was admitted to the Neonatal Intensive Care Unit, where an abdominal X-ray (AXR) revealed dilated intestines (Figure 2). The infant was given preoperative antibiotics (Ampicillin and Gentamycin).

**Figure 3:**
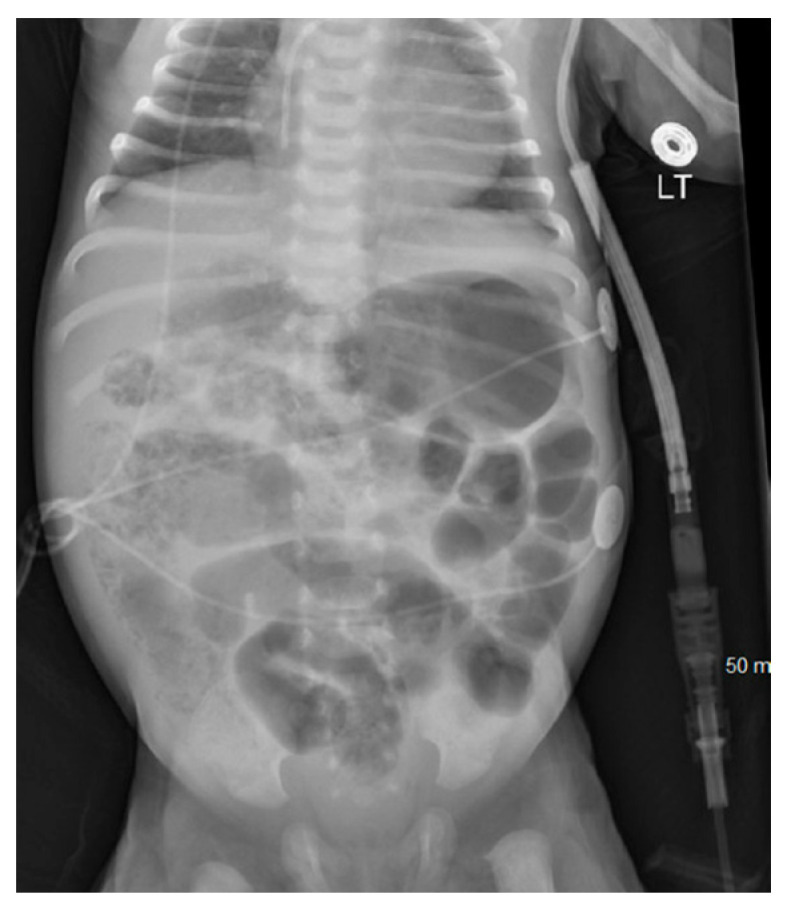
Day 25 abdominal X-ray showing pneumatosis.

At 6 h of life, exploratory laparotomy confirmed a narrow gastroschisis defect with segmental ileal and colonic atresia. The surgical intervention included a 10 cm segmental ileal resection, colo-colonic anastomosis, ileal–ileal anastomosis, and closure of the gastroschisis defect, with an initial small intestine length of 30 inches. The infant was extubated to room air by 3rd day of life (DOL), and trophic feeds started on DOL 8. The infant had culture-positive sepsis on DOL 10, requiring ceftazidime for ten days and temporary cessation of feedings. Feedings resumed on DOL 15, reaching full enteral feeds by DOL 24. On DOL 25, the infant presented with bloody stools and AXR showing dilated intestines with pneumatosis (Figure 3), indicative of necrotizing enterocolitis (NEC), prompting another period of nil-per-oral status and antibiotic therapy. Feedings restarted on DOL 32 with hypoallergenic infant formula and advanced to full feeds by DOL 37 with the infant bottle feeding all the feeds. 

**Figure 4:**
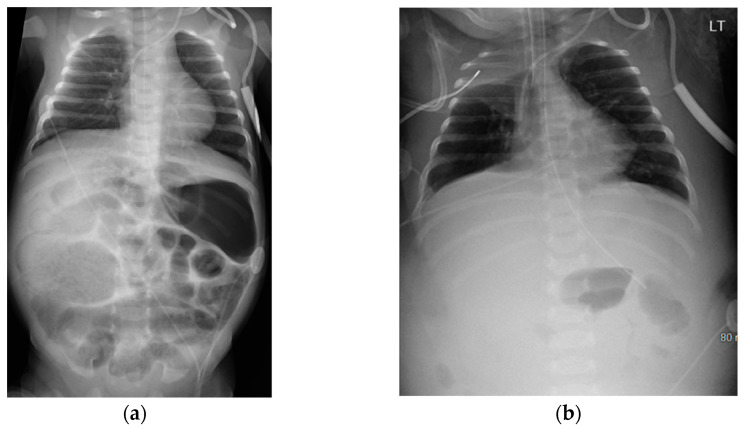
Abdominal X-ray on Day 41 and Day 50 suggesting recurrent necrotizing enterocolitis; (**a**) Day 41 abdominal X-ray revealing pneumatosis with portal venous gas; (**b**) Day 50 Abdominal X-ray revealing abdominal distension with featureless bowlet loops.

On DOL 41, the infant developed abdominal distension and tenderness, with AXR showing pneumatosis with portal venous gas (Figure 4a), suggesting recurrent NEC later progressing to featureless bowel loops (Figure 4b). The infant developed significant ascites with marked edema along with shock, necessitating a saline bolus, dopamine, multiple transfusions, and antibiotics (Vancomycin, Piperacillin-Tazobactam, and Metronidazole). An exploratory laparotomy on DOL 51 involved abdominal washout, drainage, and temporary closure of perforated intestinal loops. Due to extensive inflammatory phlegmon, double-barrel intestinal stomas were created, and the abdomen was left open for healing and covered with a sterile dressing under a transparent adhesive film, with the repaired loop positioned beneath. Wound cultures from ex-lap grew Klebsiella, leading to a change in antibiotics to Meropenem.

**Figure 5:**
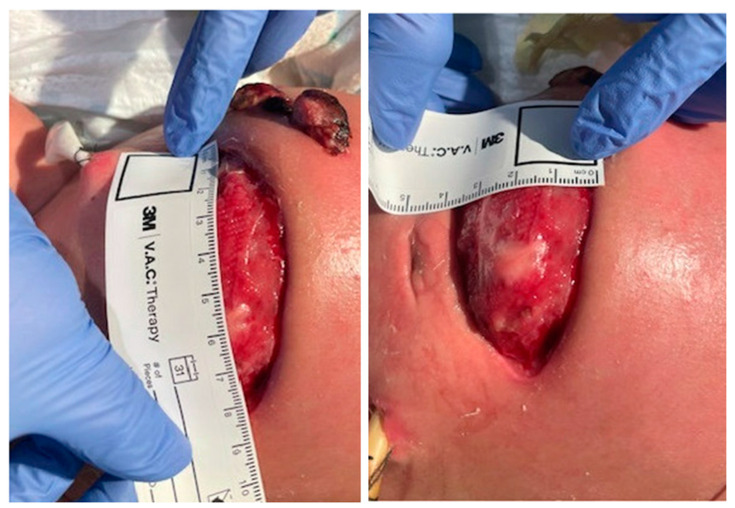
Post-ex-lap abdominal defect assessment.

On DOL 60, the infant was transferred to a Level IV Children’s Hospital for advanced surgical management and intestinal rehabilitation to manage short gut syndrome. The defect in the abdomen post-ex-lap measured 8 cm × 3.5 cm with a matted bowel without breaks. (Figure 5). A negative pressure wound therapy with vacuum assist was placed on the abdominal defect and changed every five days, along with antimicrobial foam dressing on stoma sites. The infant remained on the nasogastric sump tube to low continuous suction. The infant completed a 21-day course of Meropenem. 

On DOL 69, the infant was extubated to a high-flow nasal cannula and later weaned to room air. On DOL 75, the upper GI contrast study showed obstruction at the proximal jejunum. The infant was continued on total parenteral nutrition and SHAM feeds with hypoallergenic infant formula, which started on DOL 81 at 10 mL once daily, and slowly increased to thrice daily by DOL 106. On Day 83, the drainage catheter was removed. Palliative care managed withdrawal from prolonged narcotics and anxiolytics with methadone, tapering off midazolam, fentanyl, and dexmedetomidine over 30 days. 

The negative pressure wound therapy was stopped on DOL 92, and dressing with antibacterial polyurethane foam was initiated. On DOL 117, an echocardiogram revealed persistently depressed left ventricular function, with a left ventricular ejection fraction of 54%, and intravenous enalapril was commenced. On DOL 129, exploratory laparotomy revealed extensive adhesions and proximal jejunal obstruction near the ligament of Treitz, requiring extensive lysis of adhesions and resection of the proximal stenotic bowel, jejunum to duodenum anastomosis, enterotomy repair, and closure of ventral hernia. The intact small intestine measured 73 cm, with the presence of an ileocecal valve. 

On DOL 130, the patient was transferred to the gastrointestinal service for intestinal rehabilitation to manage short bowel syndrome (SBS). Enteral feedings via a nasogastric tube were initiated alongside parenteral nutrition (PN). Once the infant achieved a stable nasogastric feeding regimen (30 mL/h) with PN supplementation, the infant was discharged home on DOL 188 with continuous nasogastric feeds, PN supplementation, and medication for depressed left ventricular function. Long-term follow-up was planned to monitor growth, development, and cardiac function. At nine months old, the infant has a gastrostomy tube, no central line, and is on enalapril with close cardiology follow-up.

## 3. Discussion

Vanishing gastroschisis, a rare but dreaded complication with a high mortality rate (10 to 70%), occurs when the abdominal wall closes around the herniated bowel, leading to strangulation [4]. It is hypothesized to result from exposure to digestive compounds in the amniotic fluid or mesenteric constriction, leading to complications such as infarction, ischemia, atresia, stricture, or resorption, potentially leading to short bowel syndrome (SBS). Peronne et al. classified the closing or vanishing gastroschisis into four types: Type A has an ischemic bowel constricted at the ring without atresia; type B has an intestinal atresia having an ischemic but viable bowel; type C has a closing ring with non-viable external bowel, sometimes with atresia; and type D, characterized by a completely closed defect with minimal or no external bowel tissue [5]. In this case report, our patient was classified as having Type B closing gastroschisis. 

Antenatal ultrasound scans can often diagnose gastroschisis as early as 12 weeks gestation, along with elevated levels of maternal serum alpha-fetoprotein [6]. Pro-inflammatory cytokines in the amniotic fluid may lead to spontaneous preterm labor in about 30 to 40% of gastroschisis cases. Clinical evaluation typically guides delivery around 37 to 38 weeks, depending on the extent of intestinal injury [7]. Although a planned cesarean section is generally not recommended, it is worth noting that the mode of delivery does not significantly impact overall mortality or complications [8]. 

In the delivery room, washing the exposed bowel with warm saline and containing it in a sterile plastic bag is crucial to prevent additional harm. Positioning the infant in the right lateral decubitus position helps reduce abdominal pressure on the herniated bowel. Avoiding aggressive fluid resuscitation or excessive maintenance fluids is crucial to prevent the worsening of abdominal distension. Additionally, empiric sepsis evaluation and antibiotic use are unnecessary since infants with gastroschisis often exhibit elevated C-reactive protein and an elevated immature-to-total neutrophil ratio [9]. In simple reducible cases, primary reduction may entail either sutured or suture-less immediate closure, while for simple but irreducible conditions, silo reduction followed by delayed closure is often preferred. The management approach for complex gastroschisis should be individualized, considering factors like atresia or necrosis, with possible strategies including silo placement, primary closure, or ostomy creation [10].

In gastroschisis cases, necrotizing enterocolitis (NEC) occurs in 14% of simple cases compared to 8% in complex cases, as reported in a meta-analysis by Bergholz et al. [11]. Additionally, post-closure NEC rates are noted to be 6% in simple gastroschisis cases and higher at 14% in complex cases, as reported by Dekonenko et al. [12]. Our patient’s recurrent NEC and subsequent pelvic phlegmonous lesions highlight the need for advanced wound care. Negative pressure wound therapy (NPWT) has proven effective in such scenarios, promoting wound healing by reducing edema and supporting granulation tissue formation [13]. 

SBS remains a significant risk in complex gastroschisis cases, particularly in those with less than 75 cm of small intestine. Patients with an ileal remnant generally fare better than those with a jejunal remnant, and an intact ileocecal junction enhances functional capacity [14]. SBS management involves surgical, medical, and rehabilitative interventions in three phases: acute, adaptive, and maintenance. Patients often require intravenous nutrition in the acute phase, typically lasting weeks. In the acute phase, intravenous nutrition is essential, and SHAM feeding, which combines oral feeding followed by nasogastric tube suction, helps expedite oral feeding [15]. The adaptive phase, lasting up to two years or more, introduces oral or tube feedings with continued parenteral support. The intestinal rehabilitation protocol includes cycling PN for 16–20 h per day and monitoring complications such as catheter-related infections, intestinal failure-associated liver disease, fluid and electrolyte imbalances, micronutrient deficiencies, metabolic bone diseases, and small bowel bacterial overgrowth. Weekly assessments cover blood counts, basic metabolic panels, liver function, and urine electrolytes, with additional evaluations of cholesterol, copper, zinc, selenium, and essential fatty acids every three months. Maintaining urinary sodium above 30 Meq/L is crucial for optimal nutritional support [16].

The management of complex gastroschisis, particularly cases involving vanishing gastroschisis and subsequent short bowel syndrome (SBS), underscores the need for tailored, multifaceted approaches. Novel insights from our case suggest that the early and precise classification of vanishing gastroschisis can significantly guide management strategies, enhancing outcomes by addressing specific complications like ischemia or atresia more effectively. Advanced wound care techniques, such as negative pressure wound therapy (NPWT), prove critical in managing recurrent necrotizing enterocolitis (NEC) and severe abdominal complications, highlighting the need for continued research into optimizing these interventions. Future research should focus on refining these protocols and exploring new early diagnosis and intervention methods, potentially improving long-term outcomes and quality of life for affected infants. Enhanced understanding and application of these insights can drive better clinical practices and contribute to advancing neonatal surgical care.

## 4. Conclusions

Managing neonatal gastroschisis requires a comprehensive approach involving multidisciplinary collaboration. Vanishing gastroschisis, a severe complication with high mortality, necessitates an individualized, multidisciplinary approach, including vigilant monitoring for complications like NEC and SBS. The use of negative pressure wound therapy and a structured intestinal rehabilitation protocol, including cycling PN and maintaining adequate urinary sodium levels, are critical components in managing and improving outcomes for complex gastroschisis cases with SBS. Via a case study and comprehensive review, we highlight the challenges associated with gastroschisis and advocate for a tailored approach to optimize outcomes and quality of life for affected infants. Vigilant monitoring and long-term follow-up are essential to address complications and ensure comprehensive care throughout the patient’s lifespan.

## Data Availability

The original contributions presented in the study are included in the article; further inquiries can be directed to the corresponding author.
